# Hydrosilylation of Reactive Quantum Dots and Siloxanes for Stable Quantum Dot Films

**DOI:** 10.3390/polym11050905

**Published:** 2019-05-18

**Authors:** Changmin Lee, Eunhee Nam, Woosuk Lee, Heeyeop Chae

**Affiliations:** 1 School of Chemical Engineering, Sungkyunkwan University (SKKU), Suwon 16419, Korea; cm2nara@naver.com (C.L.); neh217@skku.edu (E.N.); ws3379lee@naver.com (W.L.); 2 Sungkyunkwan Advanced Institute of Nanotechnology (SAINT), Sungkyunkwan University (SKKU), Suwon 16419, Korea

**Keywords:** quantum dot, ligand exchange, hydrosilylation, siloxane, color conversion

## Abstract

The reactive acrylate-terminated CdZnSeS/ZnS quantum dots (QDs) were designed and prepared by the effective synthetic route to bond with a siloxane matrix via hydrosilylation. The conventional QD with oleic acid ligands does not have any reactivity, so the QDs were functionalized to assign reactivity for the QDs by the ligand modification of two step reactions. The oleic acid of the QDs was exchanged for hydroxyl-terminated ligands as an intermediate product by one-pot reaction. The hydroxyl-terminated QDs and acrylate-containing isocyanates were combined by nucleophilic addition reaction with forming urethane bonds and terminal acrylate groups. No degradation in quantum yield was observed after ligand exchange, nor following the nucleophilic addition reaction. The modification reactions of ligands were quantitatively controlled and their molecular structures were precisely confirmed by FT-IR and ^1^H-NMR. The QDs with acrylate ligands were then reacted with hydride-terminated polydimethylsiloxane (H-PDMS) to form a QD-siloxane matrix by thermal curing via hydro-silylation for the first time. The covalent bonding between the QDs and the siloxane matrix led to improvements in the stability against oxygen and moisture. Stability at 85 °C and 85% relative humidity (RH) were both improved by 22% for the QD-connected siloxane QD films compared with the corresponding values for conventional QD-embedded poly(methylmethacrylate) (PMMA) films. The photo-stability of the QD film after 26 h under a blue light-emitting diode (LED) was also improved by 45% in comparison with those of conventional QD-embedded PMMA films.

## 1. Introduction

Quantum dots (QDs) have received significant attention and have been widely studied throughout the last two decades due to their excellent properties, including a controllable bandgap, high emission efficiency, and narrow emission line width [1,2,3,4,5]. QDs have been studied for their use in various applications, such as photo-detectors, solar cells, bio imaging, and light emission [6,7,8,9,10,11,12,13,14]. They have been successfully commercialized as QD colour conversion films in liquid crystal display (LCD) panels, having merits of high colour purity and a wide colour gamut [15]. However, QDs are known to be degraded by oxygen, moisture, heat, and light [16,17,18,19]. Currently, inorganic barrier films are utilised to protect QD films against oxygen and moisture under ambient atmospheric conditions [20]. However, these barrier films make the fabrication process more complicated and costly, therefore, many researchers have focused their efforts on improving the stability of QD films via various approaches [21,22,23,24,25]. In most of the QD films reported thus far, including commercial films, QDs are not reactive and are randomly embedded in polymer matrices without any chemical bonding to the polymer matrices [26,27,28]. Reactive groups on the surfaces of QDs are essential to induce chemical bonding with polymers. Ligand is the only organic material in composites of QDs, and it is relatively easier to modify for functionalization of the QD than the other inorganic composites. Ligand exchange is a powerful technique, not only to impart reactivity to QDs, but also to enhance the solubility of QDs in solution and to induce cross-linking in QD films [29,30,31,32]. Although some researchers have reported surface treatment of QDs via ligand exchange, the optical properties of QDs were degraded during the ligand exchange process [33,34,35,36]. Moreover, most reports showed only schematic structures, but the structure of the modified QD could not be accurately verified using spectroscopic techniques [37,38,39,40]. Therefore, for various applications, it is strongly desired to develop a method for quantitatively functionalizing a QD ligand without deteriorating the QD in various application fields.

In this study, we presented a novel effective functionalization of QDs forming controllably acrylate terminated QDs via nucleophilic addition of hydroxyl terminated QDs as the intermediate products for the first time. The structure and substituted ratio of the acrylate functional groups were precisely verified by FT-IR and ^1^H-NMR analyses. The acrylate-functionalized QDs were then reacted with siloxanes and fabricated to the film by hydrosilylation. The thermal and moisture stabilities of resultant QD films were investigated at 85 °C and 85% relative humidity, and photo stability was evaluated under a high-flux blue light-emitting diode (LED).

## 2. Experiment

For the CdZnSeS/ZnS QD synthesis, 0.14 mmol of cadmium acetate, 3.41 mmol of Zinc acetate, and 7 mL of oleic acid were mixed in 15 mL 1-octadecene for 30 min under N_2_ flow. The mixture was heated to 110 °C and degassed under vacuum for 1 h to remove water, oxygen species, and acetic acid. The reactor charged with the mixture was then backfilled with N_2_ and the temperature was further increased to 310 °C. Selenium trioctylphosphine and sulfur trioctylphosphine were prepared by mixing 2.75 mmol of Se and 1.65 mmol of S in 2 mL of trioctylphosphine. The mixture of the Se and S precursors was rapidly injected to the reactor and then stirred vigorously for 10 min. A solution of S in 1-octadecene (0.05 mmol of sulfur in 2.4 mL of 1-octadecene) was injected to the reactor and stirred for an additional 10 min. Zinc oleate was obtained by mixing 2.9 mmol of Zinc acetate and 2 mL of oleic acid in 8 mL of 1-octadecene at 110 °C under vacuum for 1 h, and was then injected into the reactor. A thiol mixture composed of 4 mL of 6-mercaptohexanol and 8 mL of 1-octanethiol was added to the reactor and left to react for 2 h. The reaction solution was precipitated by anhydrous ethanol to powders of the QDs. The collected hydroxyl-terminated QDs (QD-OH) were dispersed in 40 mL of toluene. 4.1 g of 2-isocyanatoethyl acrylate was added to the reactor and stirred for 2 h at 30 °C for the nucleophilic addition with acrylate groups, and the acrylate-terminated QDs (QD-Acrylate) were isolated by the precipitation method. Conventional oleic-acid-coordinated QDs (QD-OA) were synthesised using the same procedure as above, but without the addition of the thiol mixture.

Four different types of QD films were fabricated and characterised in this work, as summarised in Table S1. The first QD film was formed by hydro-silylation of the QD-Acrylate with siloxane precursors (QD-Acrylate bonded to siloxane). The second QD film was fabricated from QD-OA and siloxane precursors (QD-OA in siloxane) without forming covalent bonds between the QDs and the film matrix. For the third and fourth type of QD film, QD-Acrylate and QD-OA were randomly embedded in poly(methylmethacrylate) (PMMA), respectively, (QD-Acrylate in PMMA and QD-OA in PMMA). The four mixtures were spin-coated on 4 × 4 cm Polyethylene naphthalate (PEN) substrates and glasses and cured at 140 °C for 10 min and all films had 2 μm of thicknesses, as presented in Figure S1.

## 3. Results and Discussion

**Figure 1:**
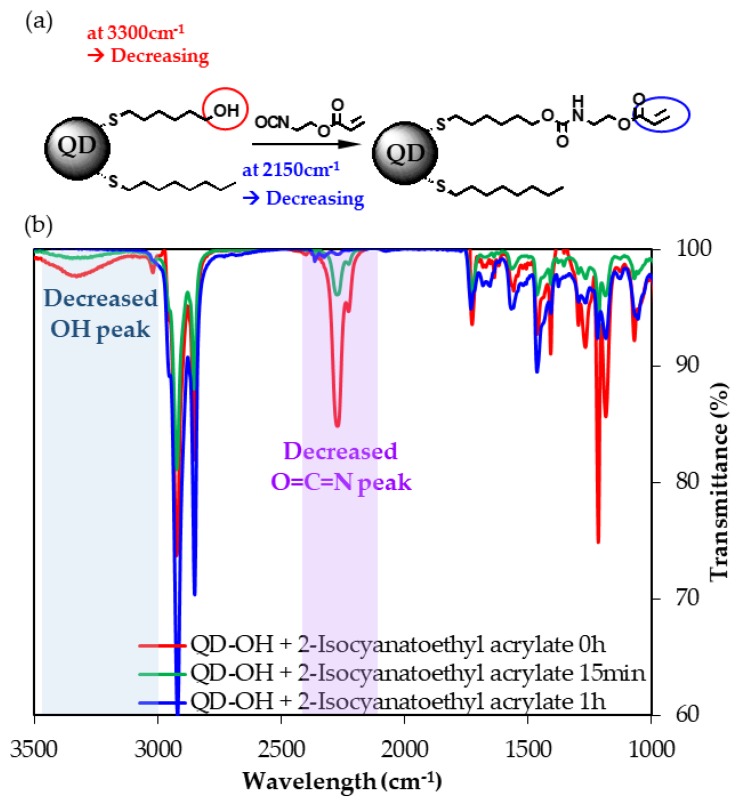
(**a**) The reaction scheme. (**b**) The reaction tracing by FT-IR. As the reaction progressed, the alcohol peak at 3300 cm^−1^ and the isocyanate peak at 2250 cm^−1^ progressively diminished in intensity.

The novel QD-Acrylate with reactive ligands was prepared via two synthetic steps. Ligand exchange was carried out without extra isolation of the QD after the QD synthesis step to introduce the terminal hydroxyl groups with one-pot reaction. The terminal hydroxyl groups were reacted with isocyanate groups for adduction of the terminal acrylate group, as illustrated in Figure 1a. This nucleophilic addition reaction proceeded to completion in 2 h at 30 °C, indicating that the terminal hydroxyl groups were sufficiently reactive to the isocyanate group of 2-isocyanatoethyl acrylate under mild conditions. Hydrophilic 6-mercaptohexanol was chosen as the source of a hydroxyl group and was mixed with hydrophobic 1-octanethiol to prevent precipitation of the QDs. The polarity of the QDs can thereby be controlled by adjusting the ratio of hydrophobic 1-octanethiol to hydrophilic 6-mercaptohexanol. If the QDs were too hydrophilic by using larger amounts of 6-mercaptohexanol than the optimized ratio, they would be inhomogeneous by precipitation of the some QDs during the reaction. It was found that QDs precipitated when 6-mercaptohexanol was used in proportions above 38% in the ligand exchange process, corresponding to a 6-mercaptohexanol:1-octanethiol ratio of 1.00:1.58. The QD-OH were isolated and reacted with 2-isocyanatoethyl acrylate to form acrylate groups on the QDs. The disappearance of the hydroxyl groups after this reaction was confirmed by the extinctions of the broad OH peak around 3300 cm^−1^ and the isocyanate peak at 2300 cm^−1^ in the FT-IR spectra, as shown in Figure 1b.

**Figure 2:**
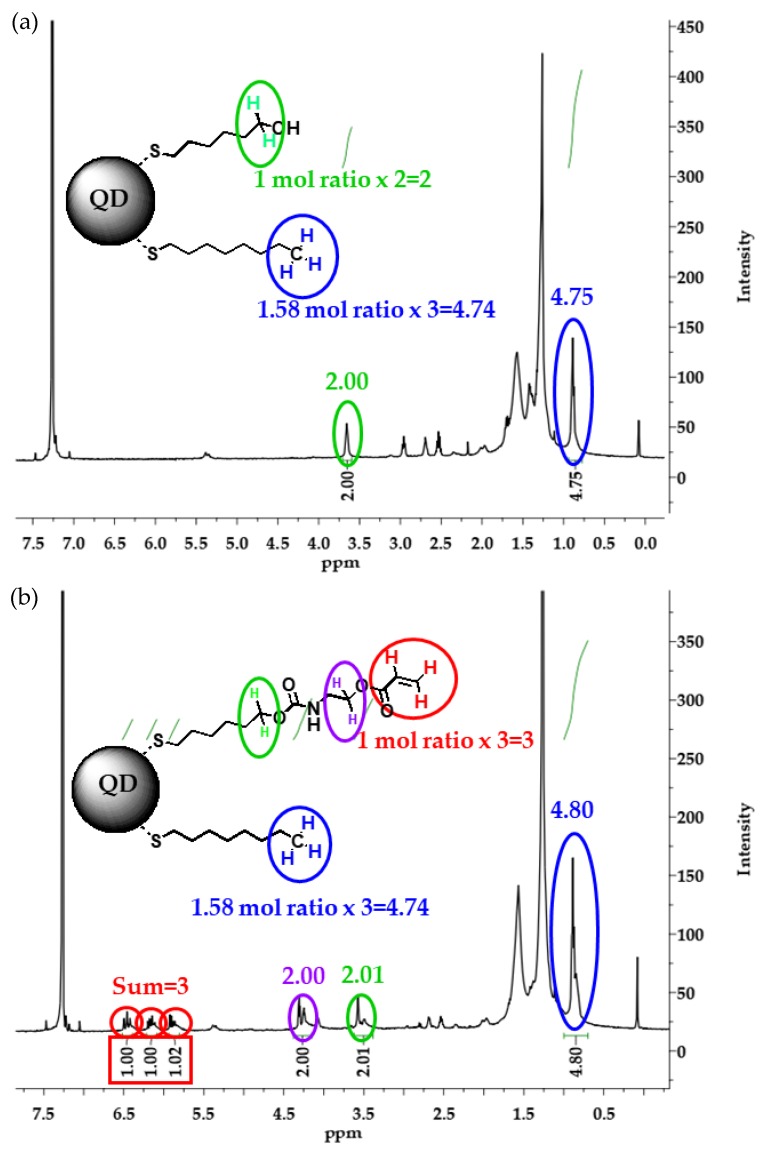
^1^H-NMR spectra of (**a**) the ligand-exchanged hydroxyl-terminated quantum dots (QD-OH) and (**b**) the acrylate-terminated quantum dots (QD-Acrylate).

The ligand structures and the substituted ratio of the hydroxyl-terminated intermediate QD-OH were characterised by evaluating ^1^H-NMR peaks of three protons at the terminal carbon of 1-octanethiol at 0.9 ppm, and of two protons adjacent to the terminal oxygen of 6-mercaptohexanol at 3.6 ppm, as shown in Figure 2a. The integrated ratio of the protons of 1-octanethiol to the protons of 6-mercaptohexanol was determined to be 2.00:4.75, and this ratio was then assessed according to the initial molar ratio of 1-octanethiol to 6-mercaptohexanol. The peak ratio determined experimentally from the ^1^H-NMR spectra was close to the molar ratio of the reactants, indicating that the ligand ratio can be controlled quantitatively by controlling the proportions of the reactants.

The structure of the QD-Acrylate was also verified by ^1^H-NMR, as shown in Figure 2b. The three protons of the terminal carbon originating from 1-octanethiol were observed at 0.9 ppm, similar to Figure 2a. The three protons of the double bond of acrylate were detected at 6.0 ppm, two protons on the neighbouring carbon of the acrylate group at 4.2 ppm, and two protons on the neighbouring carbon of the urethane group at 3.5 ppm. The integrated ratio of the proton peaks at 0.9 ppm, 4.2 ppm, and 6.0 ppm was 3.00:2.00:4.80. This ratio evidences not only that the exchange of the hydroxyl-terminated ligand proceeded quantitatively, but also that the hydroxyl groups were functionalised completely with the acrylate groups in the subsequent nucleophilic addition, as summarized in Table 1. The schematic cartoon of the synthetic route and structure is presented in Figure S2.

Optical characteristics of the QDs are summarised in Table 2, and photoluminescence (PL) and absorption spectra are presented in Figure S3. The three types of QD have the same spherical shape with the same radius of 14 nm and it was analyzed with transmission electron microscopy (TEM) and shown in Figure S4. Quantum yields (QYs) of the prepared QD-Acrylate, QD-OH, and QD-OA were 93%, 94%, and 91%, respectively. The QYs and full width at half maximums (FWHM) of the QD-OH and QD-Acrylate were quite close to those of the QD-OA. The stable QY indicates that no degradation of the optical properties of the QDs occurred during the ligand exchange, nor the following nucleophilic addition reaction. This novel synthetic method involving the one-pot ligand exchange and subsequent nucleophilic addition is quantitatively controllable without causing any degradation to the optical properties of the QDs. Although previous studies have tried to change characters of QDs by surface modification, no report precisely defined the structures of modified QDs and prevented the degradation of quantum efficiencies [41,42,43,44,45,46]. This modification method of QDs can produce high purity QDs with reproducibility, as shown in the ^1^H-NMR result, and can be extended for various functionalizations by changing functional groups in the isocyanate. We thus believe that the proposed method can be effectively applied to functionalize QDs with various moieties.

**Figure 3:**
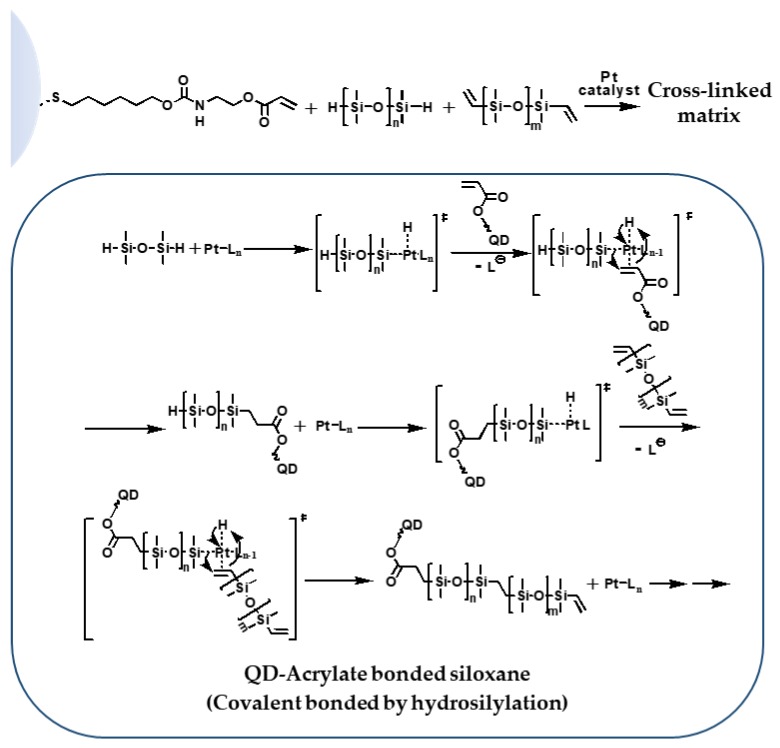
Schematic diagram of the hydrosilylation reaction between the functionalised QDs and the siloxanes in the presence of the platinum catalyst.

Siloxane resins are already commercialized as encapsulation matrices, including phosphor in light-emitting diodes (LEDs), due to their high transparency, high thermal stability, and high stability against oxygen and moisture [47]. Siloxane can be cross-linked by hydrosilylation through thermal curing of siloxane hydrides and carbon double bonds in compounds containing groups, such as vinyl, acrylate, and methacrylate [48,49,50,51]. In this work, QD-Acrylate was reacted with hydride-terminated poly(dimethylsiloxane) (H-PDMS) in the presence of a platinum (Pt) catalyst, as illustrated in Figure 3. This reaction, proposed within the Chalk–Harrod mechanism, proceeds through an intermediate platinum complex containing a hydride, a silyl ligand, and the acrylate-terminated QDs.

**Figure 4:**
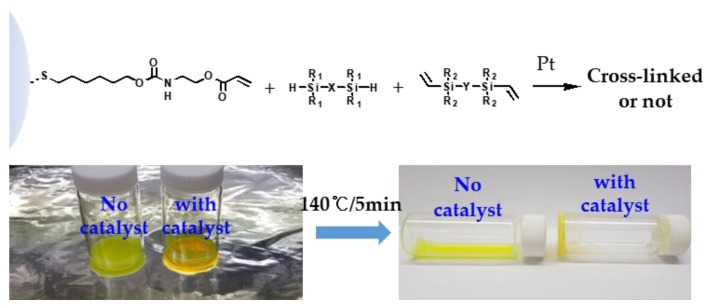
Results of the feasibility test to verify whether the functionalised QDs were reactive toward the siloxane hydrides.

The hydrosilylation reaction of the terminal acrylate groups with siloxane hydrides was verified by comparing it with the direct cross-linking reaction between QD-Acrylate and siloxane hydride in H-PDMS (Sigma-Aldrich, Mn = 580), as shown in Figure 4. Two glass vials were prepared with a mixture of QD-Acrylate and H-PDMS, where the QDs were dispersed in H-PDMS as a liquid phase. The Pt catalyst, specifically Pt_2_[(Me_2_SiCH=CH_2_)_2_O]_3_, was added to one vial while no catalyst was added to the other. The vials were cured at 140 °C, and the mixture containing the Pt catalyst was cross-linked and turned into a solid phase. The mixture without the Pt catalyst remained in the liquid phase, as seen in the left-hand vial in Figure 4.

**Figure 5:**
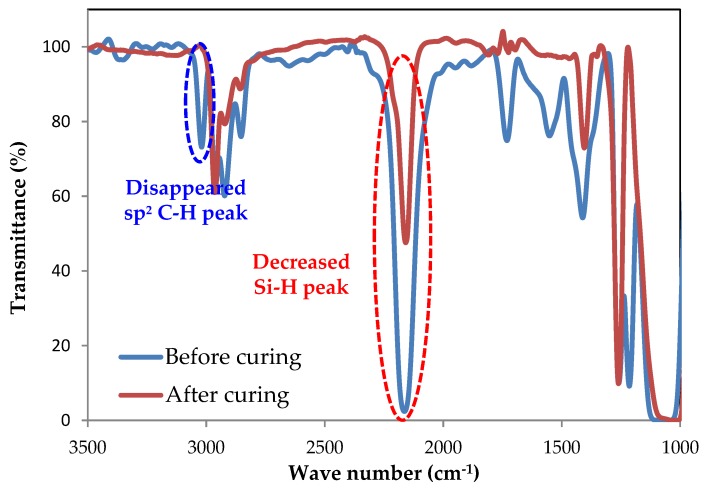
FT-IR spectra before and after the film formation by hydrosilylation. The sp^2^ C–H stretching peak at 3100 cm^−1^ from the acrylate and Si–H stretching peak at 2150 cm^−1^ from H-PDMS disappeared after the curing was finished by hydrosilylation.

The hydrosilylation reaction between the QD-Acrylate and H-PDMS was also confirmed by FT-IR spectroscopy, as shown in Figure 5. The stretching peak of the sp^2^ carbon and hydrogen in the acrylate group at 3100 cm^−1^ and the stretching peak of the Si–H bond in H-PDMS at 2200 cm^−1^ both decreased in intensity as the hydrosilylation proceeded, eventually disappearing. These results confirm that the hydrosilylation reaction between acrylate groups on the QD-Acrylate and siloxane hydride groups of H-PDMS proceeded successfully.

**Figure 6:**
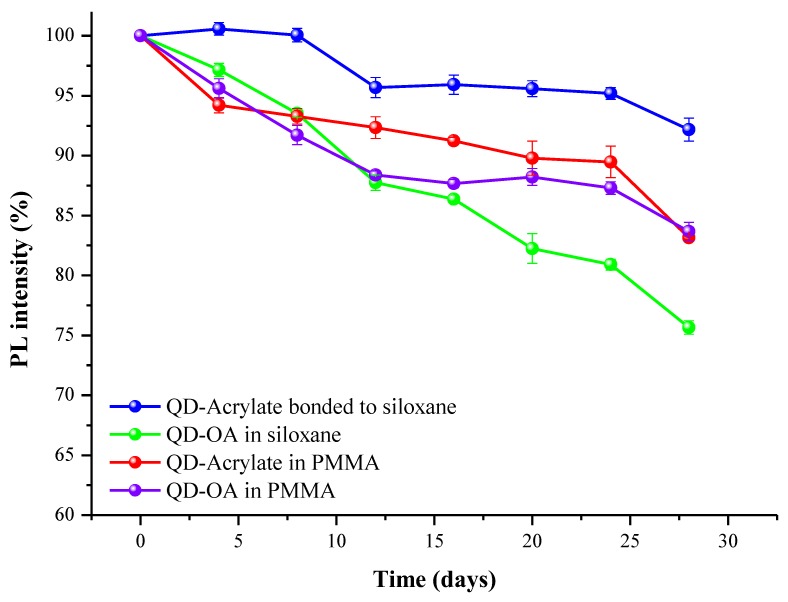
Stability test results for the film formed by hydrosilylation and for other QD samples at conditions of 85 °C and 85% relative humidity (RH), as determined by photoluminescence spectroscopy.

The moisture and heat resistances of the four QD films: QD-Acrylate bonded to siloxane, QD-OA in siloxane, QD-Acrylate in PMMA, and QD-OA in PMMA were investigated after storing the films at 85 °C and 85% relative humidity (RH) for one month. QD-Acrylate bonded to siloxane underwent an 8% drop in QY, in contrast with the 17%, 16.6% and 25% drops for QD-Acrylate in PMMA, QD-OA in PMMA, and QD-OA in siloxane, respectively. The QD-Acrylate bonded to the siloxane matrix exhibited 22% higher PL efficiency than that of QD-OA in the siloxane matrix and 11% higher PL efficiency than that of QD-Acrylate and QD-OA in PMMA films, as shown in Figure 6. (PL peaks are presented in Figure S5.) This means that the QDs randomly embedded in PMMA matrices were completely affected by the polymer matrix.

**Figure 7:**
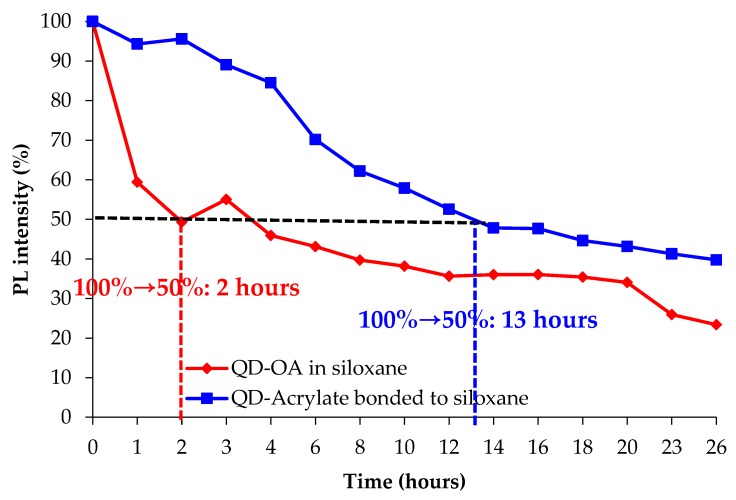
Changes in optical properties of the QD matrices during colour conversion on the blue light-emitting diode (LED).

The photo-stabilities of the films against light irradiation were determined by measuring the QY after mounting QD-Acrylate bonded to siloxane and QD-OA in PDMS on blue LEDs for colour converting blue to green emission. The QD-Acrylate bonded to the siloxane matrix showed 45% greater photo-stability than that of the QD-OA in the siloxane matrix under the blue backlight conditions, as shown in Figure 7. The QD-OA in siloxane film degraded rapidly in the early stages of the test, and then degraded slowly.

These results indicate that the QD films fabricated via the designed reaction between QDs and the siloxane matrix exhibit higher stability against harsh conditions than conventional QD matrices without covalent bonds between QDs and the matrix.

**Figure 8:**
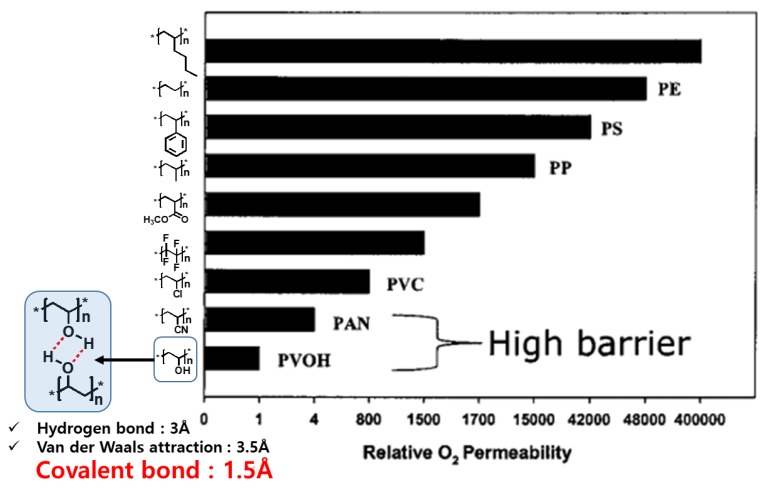
Structural effect for O_2_ barrier property in polymeric materials [52].

QDs are degraded by moisture and oxygen and the degradation is accelerated by heat and light irradiation. Thus, it is important to prevent the permeation of moisture and O_2_ into QDs, and the permeation can be blocked efficiently with control stacking distance of molecules using intermolecular interaction [52]. Advantageous structures for stacking of polymers showed lower O_2_ permeability, especially polyvinyl alcohol (PVA), which interacted by strong hydrogen bonds between the molecules, performed the high O2 barrier property with tremendous difference from the other polymers as presented in Figure 8. The QD-Acrylate was bonded to siloxanes with covalent bonds, which was a stronger interaction with closer distance between molecules, and that could prevent permeation of O_2_ and moisture to the QDs effectively. Finally, this property affected higher stability than the random QDs embedded polymer matrices. However, QDs in PDMS exhibited the worst stability among the three films. The durability of the QD-Acrylate bonded to siloxane was not attributable to the physical properties of PDMS, as PDMS is known to have an O_2_ diffusion rate 4000 times higher than that of PMMA [53,54]. The molecular weight of the used PDMS was measured to be 18,700 g/mol by gel permeation chromatography (GPC). This implies that there were only two reactive groups per approximately 246 repeat units of dimethyl siloxane in the PDMS, indicating that the cured matrix had a very low cross-linking density. The higher stability of the film of QD-Acrylate bonded to siloxane was thus attributed to covalent bonds between QDs and the siloxane matrix.

## 4. Conclusions

Acrylate-functionalized QDs (QD-Acrylate) were designed for direct bonding with siloxane precursors via hydrosilylation. QD-Acrylate was prepared in two synthetic steps including ligand exchange and a nucleophilic addition reaction. The ligand exchange proceeded via one-pot reaction from core-shell formation without requiring any extra isolation steps, and the nucleophilic addition proceeded completely with acrylate-containing isocyanates at a relatively low temperature of 30 °C. Both of modifications were carried out quantitatively and the substitution ratio can be controlled. Various functional groups having an isocyanate, even polymers, can be used to modify QDs depending on the purpose. The QD-Acrylate and the hydroxyl-terminated intermediate QD-OH were quantitatively characterised by FT-IR and ^1^H-NMR spectroscopies. No degradation in QY was observed during the ligand exchange, nor the subsequent nucleophilic addition reaction. The QD modification method developed in this work can provide a platform for the introduction of various functional groups on the surface of QDs, including via polymerisation.

The QD-Acrylate and H-PDMS were cured by heating in the presence of a platinum catalyst via a hydrosilylation reaction. QD films formed by covalent bonds between the QD-Acrylate and siloxane precursors exhibited 22% higher stability than those of QD-embedded PMMA films and QD-embedded siloxane films under conditions of 85 °C and 85% RH. This film also showed 45% higher brightness than the control group during the conversion of a blue LED backlight to green emission. The QD-siloxane matrix developed in this work can potentially be applied to QDs used for on-chip or in-chip architecture of LEDs as stable colour-conversion layers. This higher stability of the covalently bonded QD-polymer films is considered to be derived from a high density matrix on the surface of QD, sufficient in preventing the penetration of O_2_ and moisture. The acrylate-terminated QD-Acrylate can be applied not only in hydrosilylation reactions, but also in other reactions such as radical polymerisation.

## Figures and Tables

**Table 1:** The ratio of protons of the hydroxyl and acrylate terminated ligands on quantum dots (QDs).

Supplied Chemicals	6-Mercaptohexanol	2-Isocyanatoethyl acrylate	1-Octanethiol
Supplied amounts (mmol)	29.2	29.2	46.1
mol ratio	1.00	1.00	1.58
No. of protons (position)	2 (Adjacent protons to the hydroxy group)	3 (protons of the acrylate group)	3 (protons of the terminal carbon)
Integrated value (by ^1^H-NMR)	2.00 (The peak at 3.6 ppm in Figure 2a)	3.00 (The peaks from 6 to 6.5 ppm in Figure 2b)	4.75 (The peak at 0.9 ppm in Figure 2a,b)
Calculated value (by supplied chemicals)	1.00 × 2 = 2.00	1.00 × 3 = 3	1.58 × 3 = 4.74

**Table 2:** Optical properties of the QDs.

	QY * (%)	Full Width at Half Maximums (FWHM) ** (nm)	Emission _max_ (nm)
Oleic-acid-coordinated QDs (QD-OA)	91	20	525
QD-OH	94	21	527
QD-Acrylate	93	20	527

* Quantum yield; ** Full width at half maximum.

## Supplementary Materials

The following are available online at https://www.mdpi.com/2073-4360/11/5/905/s1, Table S1: Preparation of QD films by hydro-silylation; Figure S1: Thicknesses of the fabricated QD films. All of the films fabricated with 2 μm of thicknesses. (a) QD-Acrylate bonded to siloxane film (b) QD-OA in siloxane film (c) QD-Acrylate in PMMA and (d) QD-OA in PMMA film; Figure S2: Schematic cartoon of the synthetic route and structure of the modified QD. Acrylate-terminated ligands were substituted with 38.8 mol% on surface of the QD; Figure S3: (a) PL and (b) absorption spectra of QDs; Figure S4: Size and shape of QDs (TEM images) (a) QD-OA, (b) QD-OH and (c) QD-Acrylate; Figure S5: Decreasing PL peaks during storage in 85 °C/85% RH condition. (a) QD-OA embedded in PMMA matrix (b) QD-OA embedded in siloxane matrix (c) QD-Acrylate embedded in PMMA matrix and (d) QD-Acrylate bonded to siloxane matrix; Figure S6: (a) Hemisphere type PL detector and customized LED having same sized with the sample holder. (b) The stability against luminous flux proceeded together at once; Figure S7: X-ray diffraction of QD-OA. It shows typical zinc blende crystal structure of QD-OA.
