# Supplementary material for: Hydrosilylation of Reactive Quantum Dots and Siloxanes for Stable Quantum Dot Films
Source: Polymers (Basel). 2019 May 18;11(5):905. doi: 10.3390/polym11050905 (PMC6572599; doi:10.3390/polym11050905)
Supplement: Supplementary file 1 [file polymers-11-00905-s001.pdf]

Supporting Information

Hydrosilylation of reactive Quantum Dots and Siloxanes for Stable Quantum Dot Films

Changmin Lee,[a] Eunhee Nam,[a] Woosuk Lee[a] and Heeyeop Chae[a,b*]

[a]School of Chemical Engineering, Sungkyunkwan University (SKKU), Suwon, 16419, Republic of Korea

[b]Sungkyunkwan Advanced Institute of Nanotechnology (SAINT), Sungkyunkwan University (SKKU), Suwon, 16419, Republic of Korea

*Corresponding author. E-mail: Prof. H. Chae hchae@skku.edu

Materials. Cadmium acetate (99.995%), zinc acetate (99.99%), oleic acid (99%), 1-octadecene (90%), S (99.98%), 6-mercaptohexanol (97%), 1-octanethiol (98.5%) and anhydrous ethanol (99.5%), Poly(methylmethacrylate) (MW : 110,000) were purchased from Sigma-Aldrich Inc. Se (99.999%) and trioctylphosphine (90%) were purchased from Alfa Aesar. Siloxane precursors (Sylgard® 184A and B) were supplied from Dow corning and 4 × 4 cm PEN was purchased from DuPont Teijin Films. All reagents used in this study were purchased at anlytical reagent grade and used for synthesis without further purification.

Characterization. Fourier-transform infrared (FT-IR) spectroscopy (JASCO, FT-IR 4600) , ^{1}H-NMR spectroscopy at 500 MHz (Varian Technologies, Unity Inova) were used for structural analysis of the QDs. Quantum efficiency spectrometry (Otsuka Electronics, QE-2100), customized blue LED kit (DS Lab.) and voltage−current source unit (Tektronix, Keithley 2400) was employed for the color conversion and stability test. The shape and size of the QDs were verified by transmission electron microscopy (TEM) (JEOL, JEM-2100F). The thicknesses of films were measured by alpha step profiler (KLA-Tencor, P-7)

Table S1. Preparation of QD films by hydro-silylation

QD type	QD	PMMA	Sylgard 184A*	Sylgard 184B**	Toluene
QD-Acrylate	60 mg		0.4 g	0.1 g	0.3 g
QD-Acrylate	60 mg	0.5 g			5 g
QD-OA	60mg		0.4 g	0.1 g	0.3 g
QD-OA	60 mg	0.5 g			5 g

* Sylgard® silicon elastomer / Dow corning

** Sylgard® silicon elastomer curing agent / Dow corning

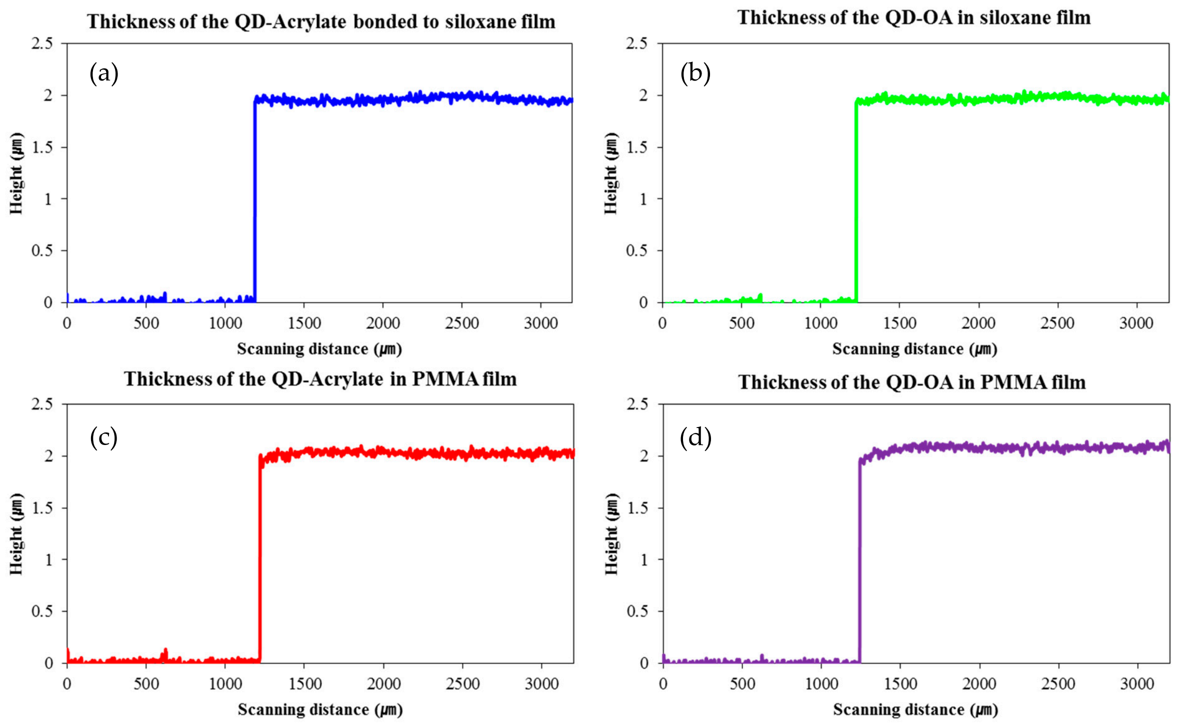

Figure S1. Thicknesses of the fabricated QD films. All of the films fabricated with 2 μm of thicknesses. (a) QD-Acrylate bonded to siloxane film (b) QD-OA in siloxane film (c) QD-Acrylate in PMMA and (d) QD-OA in PMMA film

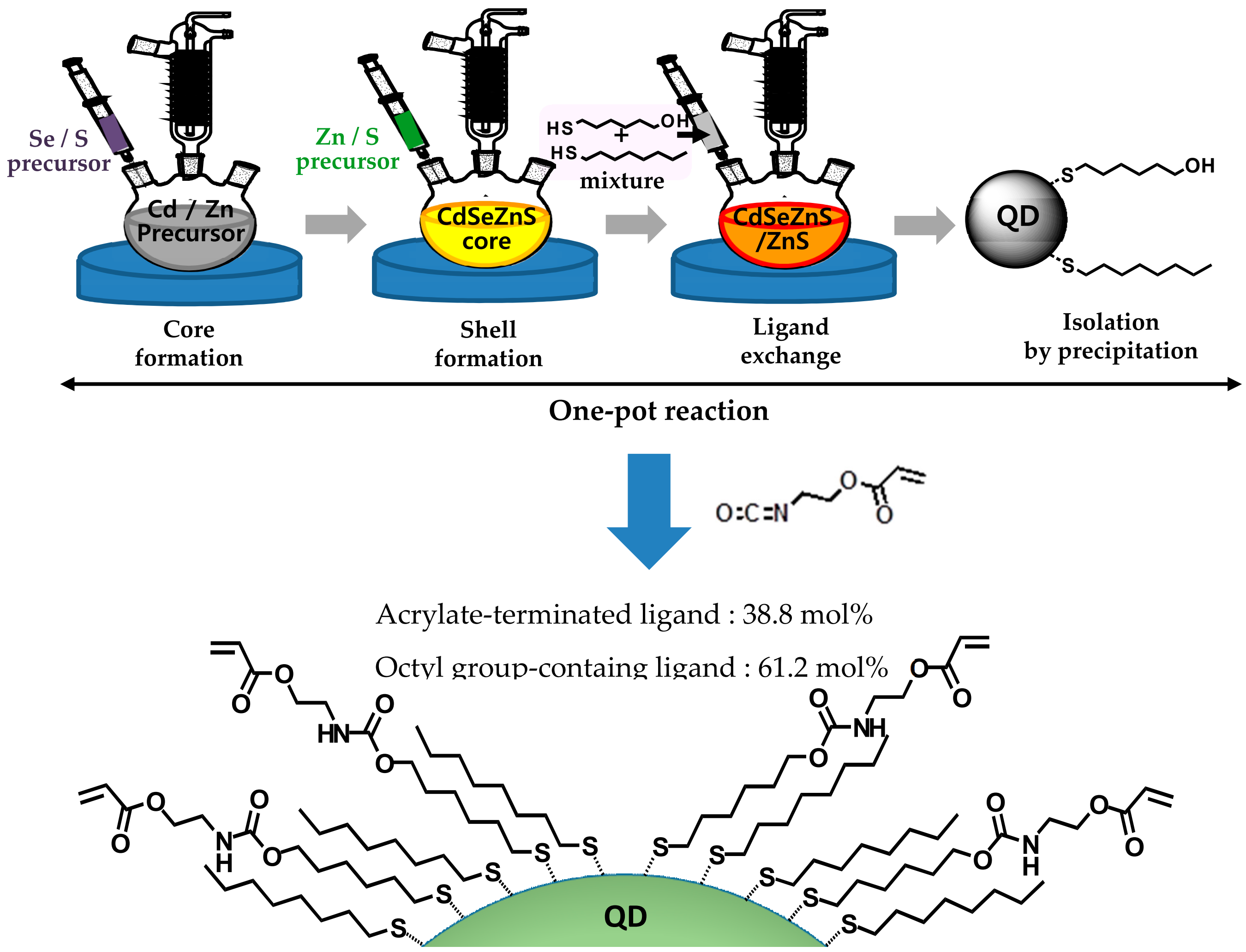

Figure S2. Schematic cartoon of the synthetic route and structure of the modified QD. Acrylate-terminated ligands were substituted with 38.8 mol% on surface of the QD.

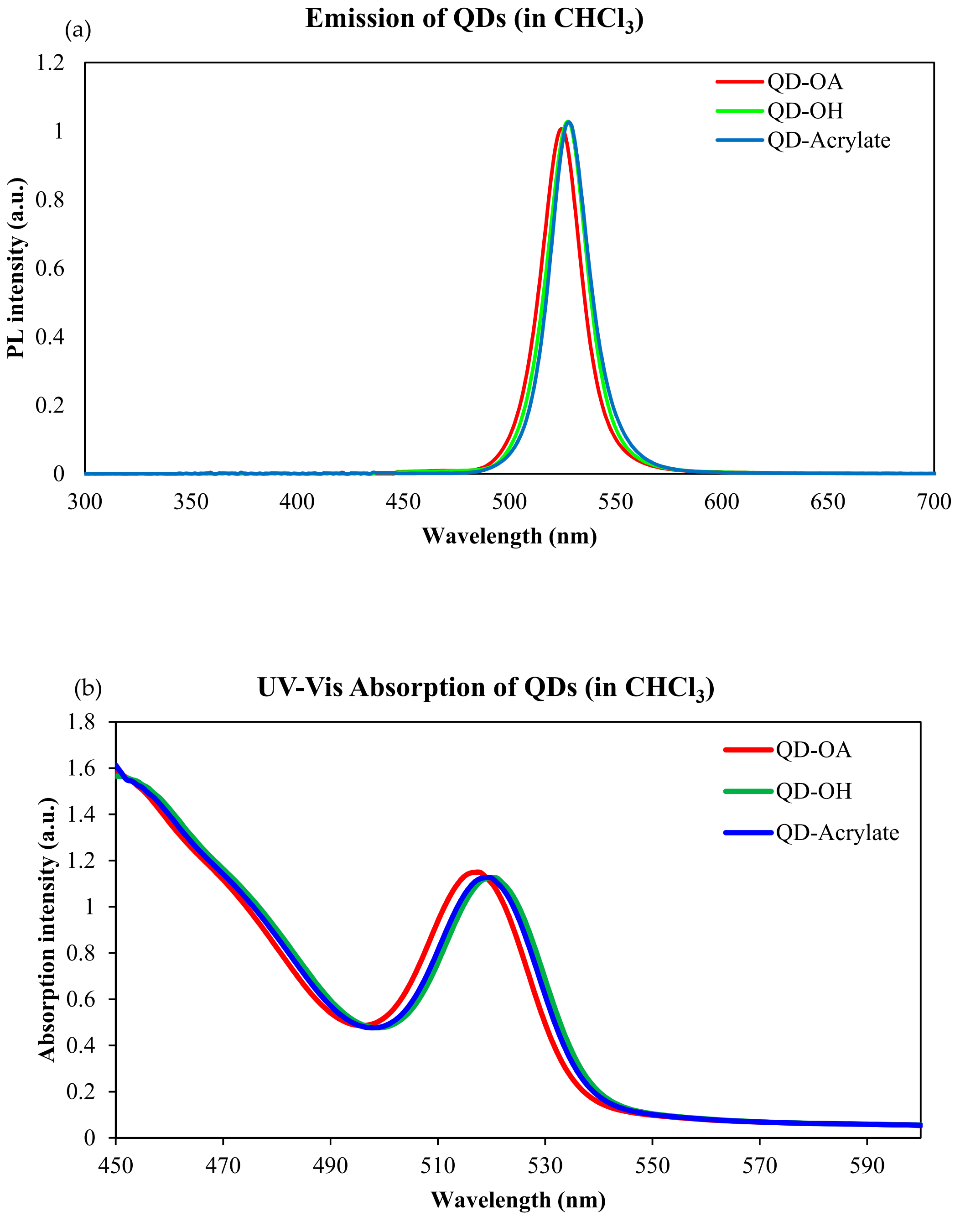

Figure S3. (a) PL and (b) absorption spectra of QDs

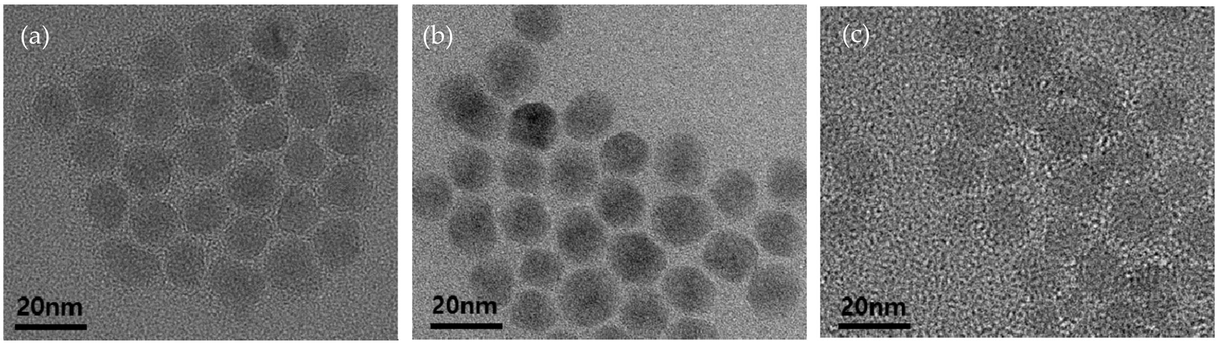

Figure S4. Size and shape of QDs (TEM images) (a) QD-OA, (b) QD-OH and (c) QD-Acrylate.

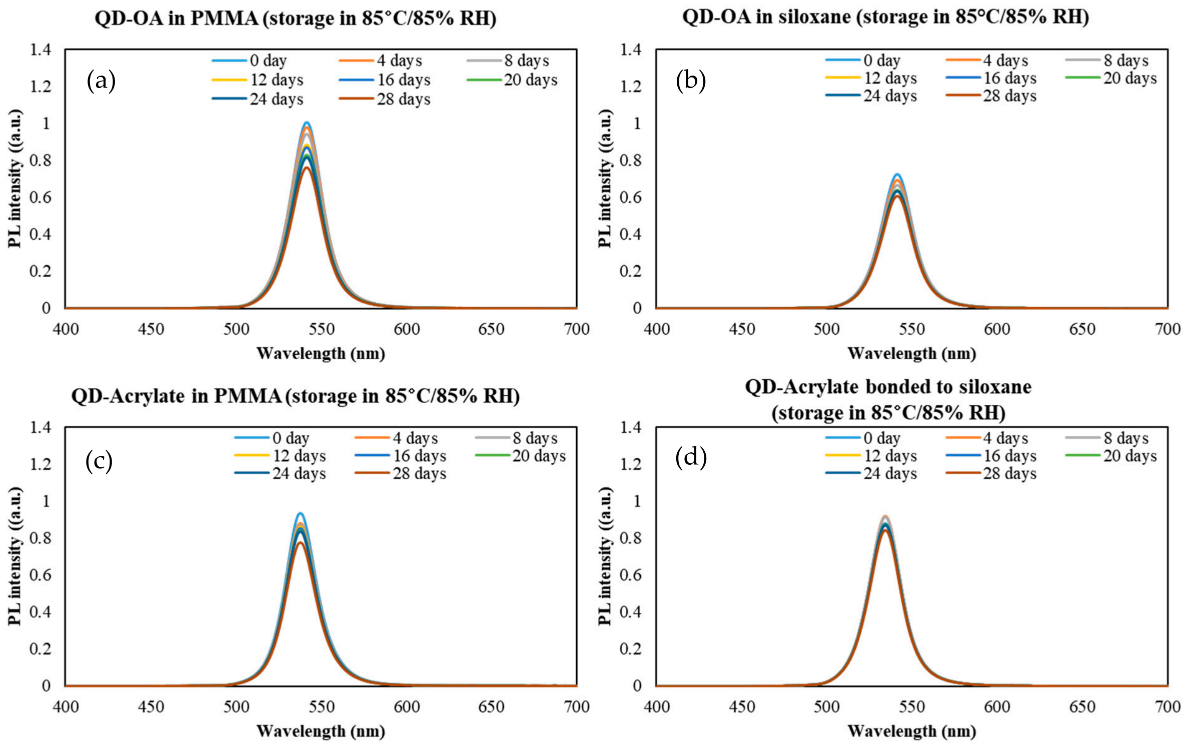

Figure S5. Decreasing PL peaks during storage in 85℃/85% RH condition. (a) QD-OA embedded in PMMA matrix (b) QD-OA embedded in siloxane matrix (c) QD-Acrylate embedded in PMMA matrix and (d) QD-Acrylate bonded to siloxane matrix.

(a)

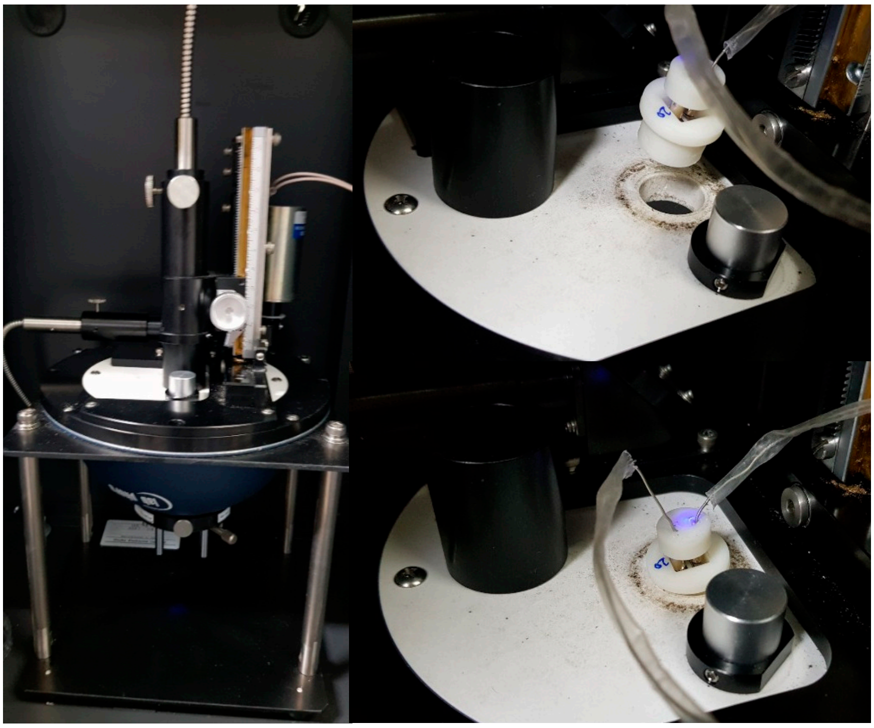

(b)

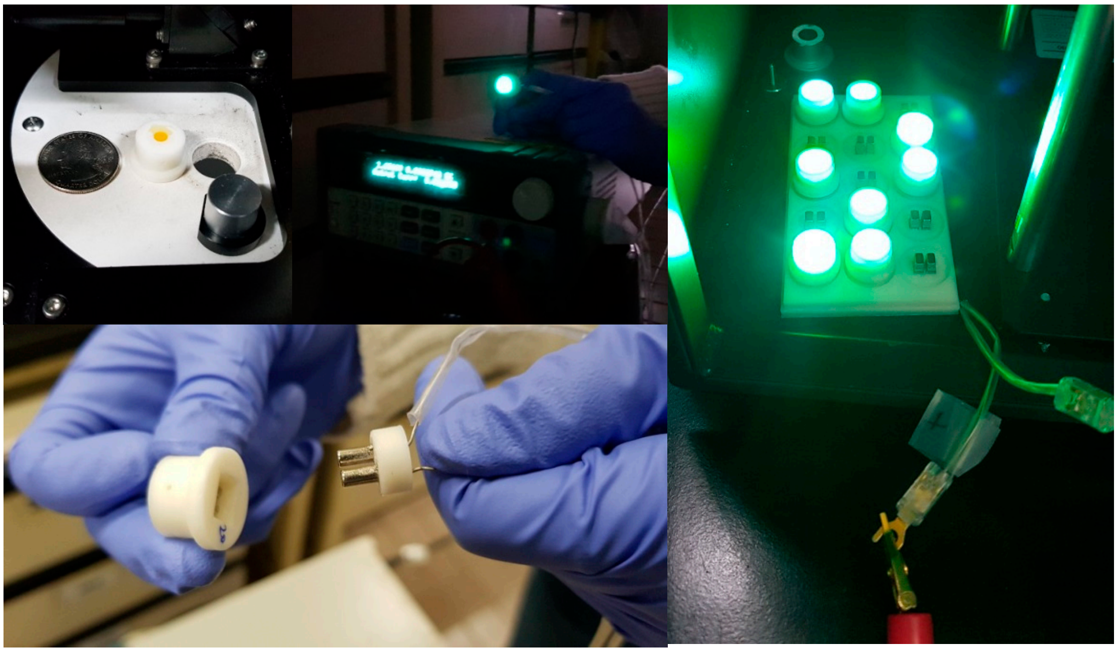

Figure S6. (a) Hemisphere type PL detector and customized LED having same sized with the sample holder. (b) The stability against luminous flux proceeded together at once.

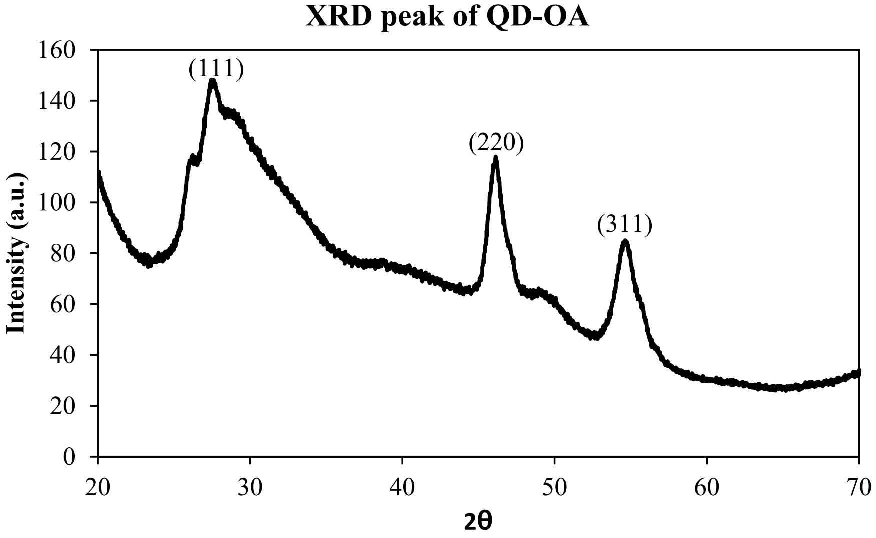

Figure S7. X-ray diffraction of QD-OA. It shows typical zinc blende crystal structure of QD-OA.
